# Superiority of 3D-DIR over 3D-FLAIR in the Detection of Cortical Lesions and Correlation with Disability in Multiple Sclerosis: A Multicenter Study

**DOI:** 10.3390/diagnostics15243103

**Published:** 2025-12-06

**Authors:** Irene Grazzini, Davide Del Roscio, Marco Cirinei, Benedetta Calchetti, Matteo Grammatico, Giulia Spossati, Lorenzo Malatesti, Teresa De Stefano, Andrea Cuneo, Sara Leonini, Ernesto Piane, Lorenzo Testaverde

**Affiliations:** 1 Unit of Neuroradiology, Department of Diagnostic Imaging, San Donato Hospital, 52100 Arezzo, Italy; 2 Unit of Neuroradiology, Department of Diagnostic Imaging, Misericordia Hospital, 58100 Grosseto, Italy; davide.delroscio@uslsudest.toscana.it (D.D.R.); marco.cirinei@uslsudest.toscana.it (M.C.); teresa.destefano@uslsudest.toscana.it (T.D.S.); ernesto.piane@uslsudest.toscana.it (E.P.); 3 Department of Neurology, San Donato Hospital, 52100 Arezzo, Italy; benedetta.calchetti@uslsudest.toscana.it (B.C.); matteo.grammatico@uslsudest.toscana.it (M.G.); 4 Department of Technical, Rehabilitation, and Preventive Health Professions, San Donato Hospital, 52100 Arezzo, Italy; giulia.spossati@uslsudest.toscana.it (G.S.); lorenzo.malatesti@uslsudest.toscana.it (L.M.); 5 Unit of Diagnostic and Therapeutic Neuroradiology, Department of Diagnostic Imaging, Azienda Ospedaliera Universitaria Senese, 53100 Siena, Italy; andreacuneo23@gmail.com (A.C.); sara.leonini@ao-siena.toscana.it (S.L.); 6 Unit of Neuroradiology, Department of Diagnostic Imaging, United Hospitals of Livorno, 57124 Livorno, Italy; doctor.lot@gmail.com

**Keywords:** multiple sclerosis, magnetic resonance, Double Inversion Recovery (DIR) sequence, demyelination

## Abstract

**Background/Objectives**: The aim of the study was to compare diagnostic performance of 3D-Double Inversion Recovery (DIR) and 3D-Fluid-Attenuated Inversion Recovery (FLAIR) sequences in the detection of brain lesions in Multiple Sclerosis (MS) patients, especially cortical ones, and to evaluate potential correlation between lesion number and clinical outcome. **Methods**: From April 2021 to July 2024, 278 MS patients (201 females, 77 males, mean age 47.01 ± 12.668 years) underwent brain MRI in three Italian Institutions using 1.5 T systems; 3D-FLAIR and 3D-DIR sequences were obtained with an identical anatomic position. Clinical disability was evaluated by the expanded disability state score (EDSS). Data analysis was performed using the Wilcoxon test for lesion count differences (primary endpoint), and Chi-square test and Spearman for EDSS correlation (secondary endpoint); a *p* < 0.05 was considered as statistically significant. **Results**: A significantly higher total number of lesions was displayed on DIR images (*n* = 6601) compared with FLAIR (*n* = 6484) (*p* < 0.001). The mean number of cortical lesions identified with DIR (1.56 ± 2.767) was significantly higher than the mean number of cortical lesions detected with FLAIR (0.52 ± 1.029) (*p* < 0.001). Conversely, FLAIR sequences detected a significantly higher mean number of subcortical lesions (9.34 ± 8.663) compared to DIR (8.94 ± 8.415) (*p* < 0.001). A significant correlation was found between EDSS and the number of juxtacortical and cortical lesions detected with DIR with a *p* < 0.001. **Conclusions**: 3D-DIR is superior to 3D-FLAIR in detecting cortical lesions, which are correlated to clinical disability, and it should be implemented for the diagnosis and prognostic evaluation in MS patients.

## 1. Introduction

Multiple Sclerosis (MS) is a chronic inflammatory demyelinating disorder that may lead to physical and cognitive disability [1,2]. Although it is known that MS mainly affects the white matter (WM), it also frequently occurs in the cortical gray matter (GM) [3,4]. Furthermore, cortical GM lesions seem to be related to disability [5,6], and previous studies suggested that the degree of disability progression in MS positively correlates with cortical lesions more than the subcortical WM load [7,8,9]. In fact, cortical lesions have been included in the recent 2017 revised McDonald’s diagnostic criteria for MS [10].

However, cortical lesions may be unapparent with conventional imaging sequences such as Fluid-Attenuated Inversion Recovery (FLAIR) [11,12]. Improved visualization of cortical lesions has been achieved with the development of double inversion-recovery (DIR) sequence [13,14]. In particular, it has been reported that DIR sequences show a higher sensitivity for cortical lesion detection compared to FLAIR sequences (*p* = 0.003 with a relative ratio of 99%) [7]. DIR is a pulse sequence with two inversion times, used in neuroimaging to suppress signal from both cerebrospinal fluid and WM [15,16,17]. Although this sequence has been introduced since 1994, currently DIR are largely absent in routine diagnostic protocols, and many studies on this topic still analyzed non-volumetric 2D-DIR sequences, which are limited by multiple artifacts [7,11,13,18,19,20,21,22].

Thus, the aim of the study was to compare diagnostic performance of 3D-DIR and 3D-FLAIR sequences in the detection of MS cerebral lesions, especially cortical ones, and to evaluate potential correlation between lesion number and clinical scores, in order to enhance 3D-DIR’s role in the management of MS patients.

## 2. Materials and Methods

### 2.1. Population

**Figure 1:**
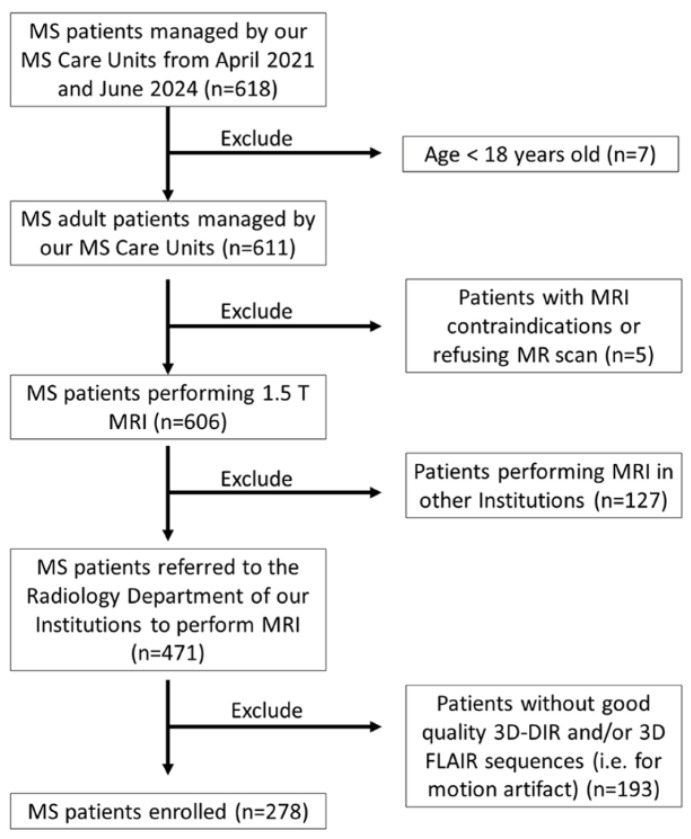
Flow chart of the patient cohort.

This prospective, multicenter study received approval from the Institutional Review Board, with written informed consent signed by all participants. The study was conducted in three Italian Institutions from April 2021 to July 2024 and included MS patients according to the following inclusion criteria: age ≥ 18 years; neurological diagnosis of MS according to the 2017 McDonald criteria; and MRI brain scan including both 3D-DIR and 3D-FLAIR sequences. Figure 1 shows the recruitment flow chart.

Physical disability was assessed with the Expanded Disability State Score (EDSS) by two experienced MS neurologists (B.C and M.G) at the time of MRI examination. EDSS is the most common clinical score currently used to assess the degree of disability in MS patients, ranging from 0 (normal) to 10 (death due to MS).

### 2.2. MR Imaging Acquisition

All patients consecutively underwent brain MRI in the Radiology Department of the three Italian Institutions using 1.5T scans (Siemens Avanto, Erlangen, Germany, or GE Healthcare Signa, Chicago, IL, USA) with a 12-channel array head coil. All patients underwent examination in the supine position, with the head oriented forward. Our standardized MR protocol for MS patients included

(a) Conventional imaging: axial T1-weighted, obtained pre- and post-intravenous injection of 0.1 mmol/kg of gadolinium-based contrast agents, axial T2WI and axial DWI.
(b) Sagittal 3D-DIR and sagittal 3D-FLAIR, obtained with identical anatomic position in each scanner and patient.

We used the following acquisition parameters for the 3D-DIR sequence:

- On GE scanner: acquisition plane 3D Sagittal, TR 6800 ms, TE 112 ms, TI 1 2650 ms, TI 2 445 ms, Refocusing Flip Angle (variable), acquisition matrix 256 × 256, reconstruction matrix 256 × 256, FOV 225, slice number 96, acquisition slice thickness 1.8, reconstruction slice thickness 1 mm, gap 0, NEX 1, parallel imaging: Autocalibrating Reconstruction for Cartesian Imaging (ARC) with acceleration factor 2; acquisition time 6 min 11 s.
- On Siemens scanner: acquisition plane 3D Sagittal, TR 7500 ms, TE 310 ms, TI 1 3000 ms, TI 2 450 ms, Refocusing Flip Angle (variable), acquisition matrix 192 × 192, reconstruction matrix 192 × 192, FOV 280, slice number 128, acquisition slice thickness 1.5 mm, reconstruction slice thickness 1 mm, gap 0, NSA 1, parallel imaging: integrated Parallel Acquisition Techniques (iPAT) Mode GeneRalized Autocalibrating Partial Parallel Acquisition (GRAPPA) with acceleration factor 2; acquisition time 5 min 39 s.

Acquisition parameters for the 3D–FLAIR sequence were

- On GE scanner: acquisition plane 3D Sagittal, TR 6000 ms, TE 105, TI 1908 ms, Refocusing Flip Angle (variable), acquisition matrix 256 × 256, reconstruction matrix ZIP 512, FOV 256, slice number 96, acquisition slice thickness 1.8 mm, reconstruction slice thickness 1 mm, gap 0, NEX 1, parallel imaging: ARC with acceleration factor 2; acquisition time 7 min 09 s.
- On Siemens scanner: acquisition plane 3D Sagittal, TR 10,000 ms, TE 372 ms, TI 2500, Refocusing Flip Angle (variable), acquisition matrix 179 × 256, reconstruction matrix 256 × 256, FOV 256, slice number 144, acquisition slice thickness 1 mm, reconstruction slice thickness 1 mm, gap 0, NSA 1, parallel imaging: iPAT Mode GRAPPA with acceleration factor 4; acquisition time 6 min 10 s.

Detailed imaging acquisition parameters are summarized in Table 1. Total acquisition time for the MS protocol was 25 min 30 s on the Siemens scanner, and 27 min 56 s on the GE scanner.

Radiological evaluations of all the obtained 3D-DIR and 3D-FLAIR sequences were performed independently by two neuroradiologists (I.G. and L.T.), blinded to clinical information. In cases of disagreement, final assessment was reached by consensus, with all sequences and the 6-month MR follow-up available; the follow-up images were used to differential between lesion confirmations versus artifacts. High signal-intensity lesions with a size of >3 mm were counted and classified according to their location as infratentorial, periventricular WM (adjacent to the lateral ventricles), subcortical WM (deep in WM), juxtacortical (in the WM but touching the cortex), and cortical (involving the cortical GM) regions. The lesion numbers in each region were determined.

### 2.3. Statistical Analysis

The statistical analysis was performed by the IBM© SPSS© Statistics version 23 (IBM© Corp., Armonk, NY, USA). Total number of detected lesions and lesions in each region were separately determined and stated as mean ± SD. Frequency and percentage were used to describe qualitative data. The Shapiro–Wilk test was employed to assess data normality. To compare the number of lesions detected by different MRI sequences, a Wilcoxon matched-pairs signed-rank test was performed (primary endpoint). The Chi-square test was calculated to examine the relationship between the number of cortical MS plaques and EDSS of the patients (secondary endpoint). Spearman correlation, with two-tailed significance, was also used to analyze the relationship between the number of MS cortical plaques and the clinical data of the patients. EDSS scores were also categorized into three groups (score 0–3: mild; score 3–6: moderate; score > 7: severe). A *p* value < 0.05 was considered as statistically significant; the results were considered highly significant with a *p* < 0.001.

## 3. Results

### 3.1. Final Study Population

The final study population included 278 patients (201 females and 77 males, mean age 47.01 ± 12.668 years, range 18–75 years). Mean EDSS score was 1.18 ± 1.687, ranging from 0 to 8. Summary of the demographics of the study is presented in Table 2.

### 3.2. Lesion Count

**Figure 2:**
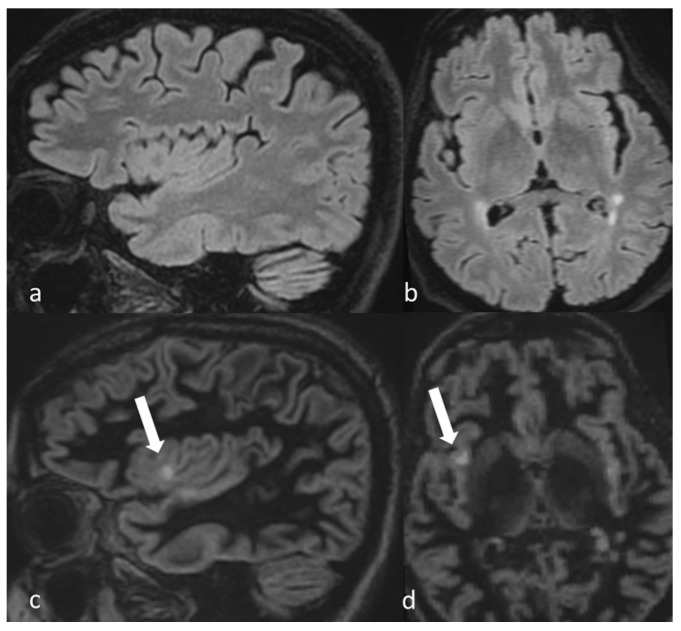
(**a**,**b**) The right insula cortical lesion (arrow) is unapparent on sagittal and axial FLAIR; (**c**,**d**) the same cortical lesion is well detected on sagittal and axial DIR images.

**Figure 3:**
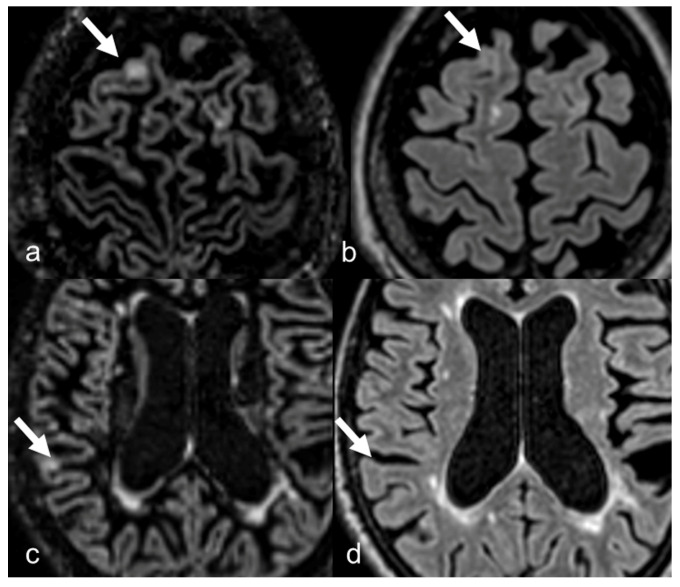
(**a**,**c**) The right cortical lesions (arrows) are well detected on DIR images; (**b**,**d**) the same lesion is unapparent on FLAIR images.

A significantly higher total number of MS lesions were detected on DIR (*n* = 6601) compared with FLAIR images (*n* = 6484) with *p* < 0.001. In particular, the number of cortical lesions detected with DIR sequences (total of 435 lesions, average of 1.56 ± 2.767) was significantly higher compared to FLAIR sequences (total 144 lesions, average of 0.52 ± 1.029), with a *p* < 0.001. On the other hand, the number of subcortical lesions detected with FLAIR (total of 2596, average of 9.34 ± 8.663) was significantly higher than the number detected with DIR (total of 2485, average of 8.94 ± 8.415) with a *p* < 0.001 (Figure 2 and Figure 3).

**Figure 4:**
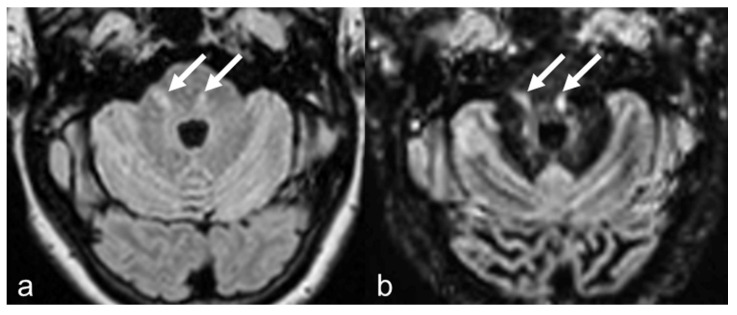
The pontine lesions (arrows) are well detected both on FLAIR (**a**) and on DIR images (**b**).

**Figure 5:**
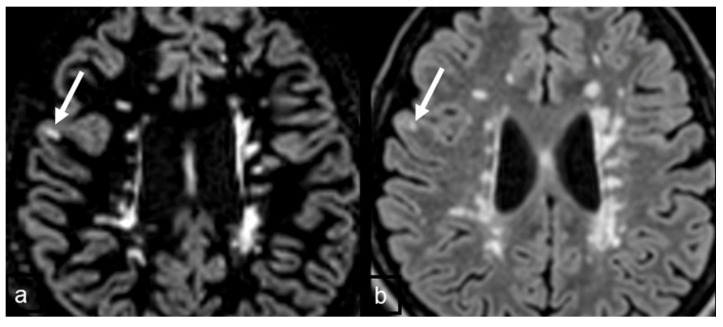
The right frontal juxtacortical lesion (arrow) is detected both with DIR (**a**) and with FLAIR images (**b**); however, it is more evident on DIR. Note that DIR and FLAIR images are similar in detection of periventricular lesions.

No notable difference was observed in the number of detected lesions between both sequences in periventricular and infratentorial regions (Figure 4). Juxtacortical lesions resulted numerically higher with DIR, but not statistically significant (Figure 4 and Figure 5). These results are summarized in Table 3. Moreover, 3D-DIR sequences were able to detect ≥1 cortical lesion in 151/278 patients (54.3%), while 3D-FLAIR only in 80/278 (28.8%).

### 3.3. Correlation with Clinical Score

**Figure 6:**
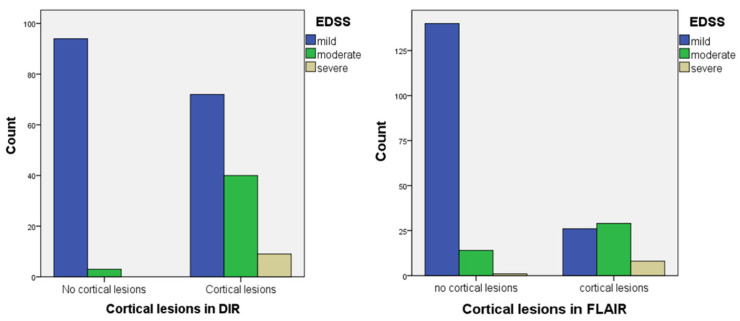
Relationship between cortical affection detected in DIR and FLAIR, and EDSS of the MS patients.

Regarding the association between the clinical EDSS groups and cortical affection, the Chi square showed a highly statistically significant association with *p* < 0.001 (χ^2^ = 41.615 in DIR, and 61.006 in FLAIR). All MS patients with severe EDSS exhibited cortical lesions in DIR in our data set (Table 4 and Figure 6).

A statistically significant positive correlation was found between EDSS values and the number of cortical lesions in 3D-FLAIR and 3D-DIR sequences (correlation coefficient 0.662, *p* = 0.001; correlation coefficient 0.874, *p* < 0.001, respectively). The relationship between the number of cortical lesions for each sequence and the EDSS score is shown in Table 5.

## 4. Discussion

The detection of cortical MS lesions was incorporated into the 2017 revisions of the McDonald criteria [10]. The current guidelines recommend identification of cortical lesions based on standard images and, optionally, on DIR or Phase-Sensitive Inversion Recovery (PSIR) [23]. Although a preliminary study affirmed that 3D-MPRAGE provided better characterization of cortical lesions [24], the same authors lately demonstrated that the combination DIR/PSIR is superior to 3D-MPRAGE for detection of cortical lesions in MS [25]. They also reported that DIR and PSIR are both 1.5–5 times more sensitive than conventional sequences in the detection of cortical lesions [20]. Favaretto et al. [26] confirmed that the parallel analysis of DIR and PSIR images might improve cortical lesion identification and classification. However, the current guidelines suggested the use of these sequences in centers with a sufficient level of expertise and standardization of image acquisition [23]. Thus, although Forslin et al. [27] reported that PSIR might be superior to DIR in detecting cortical MS lesions, we chose to analyze DIR in our study, as this sequence has been largely used and standardized in our centers since 2019 for MS and seizure protocols. Standardized and clearly defined procedures were also intended to prevent implementation bias.

Our results showed that the 3D-DIR sequence was significantly superior to 3D-FLAIR with regard to detection of overall number of MS plaques and in particular, of cortical lesions, conforming to the data from previous studies [7,11,13,17,20,21,28]. In fact, 3D-DIR was able to detect cortical lesions in 151/278 patients, compared to 80/278 with 3D-FLAIR. Thus, 3D-DIR sequence may allow a more accurate evaluation of dissemination in space of the disease according to McDonald criteria, and consequently an earlier diagnosis of MS. Abidi et al. [11] and Elkholy et al. [28] found a significantly higher total number of lesions displayed on DIR than on FLAIR images. They also found that DIR has a higher accuracy in the detection of cortical lesions (average of 7.13 ± 7.19 in DIR compared to 1.41 ± 1.76 in FLAIR, and average of 1.29 ± 1.04 lesions in DIR compared to 0.5 ± 0.71 in FLAIR, respectively). Vural et al. [13] and Elnekeidy et al. [7] also reported the highest accuracy of the DIR in the detection of cortical lesions and its enhanced ability to discriminate between mixed WM–gray matter, and juxtacortical and pure cortical lesions. A study by Ertan et al. [29] stated that the 3D-DIR sequence detected cortical lesions five times more than that of the 3D-FLAIR sequence. In a paper by Abdelrahman et al. [17], the number of cortical plaques detected by 3D-DIR sequence in 82 patients was significantly more than the 3D-FLAIR sequence with 185% percent improvement (*p* < 0.001). In Wattjes et al. [21], the DIR at 3T showed a higher number of lesions compared with the FLAIR (7% gain, *p* = 0.04) and the T2 TSE (15% gain, *p* = 0.01), and that the higher sensitivity was significant for the infratentorial region (56% gain, *p* = 0.02, and 44% gain, *p* = 0.02, respectively). However, these studies had small population cohorts (15, 55, 34, 82, 26, 32, and 24 MS patients, respectively [7,11,13,17,19,28,29]), and they reported that 3D-DIR was better than FLAIR for lesion number detection also in the periventricular, juxtacortical, and infratentorial lesions [7,11,17,21,28]. In our results, we did not find significant differences in these regions, and the 3D-FLAIR was superior to 3D-DIR in the detection of subcortical plaques, according to data from Vural et al. [13]. It could be due to different scanners we used, GE and Siemens, while the other studies were performed on Philips systems [7,11,13,17,19,20,21,22,28], with consequently different vendor parameters. However, Vural et al. [13] obtained similar results using a Philip Achieva system, too. We also used 1.5 scanners, as many previous papers [7,11,13,17,19,22,28], whilst Nelson et al. [20] and Wattjes et al. [21] utilized 3T scanners. As Siemens and GE systems have been scarcely applied in the literature on this topic, further studies are required to assess whether different vendor scanners have an impact on regional findings.

We observed an association between cortical lesions and EDSS, as described in the previous literature [9,13,17,19,22]. This association was evident with both DIR and FLAIR imaging, though DIR demonstrated stronger statistical significance. Nonetheless, our findings do not confirm a significant advantage of DIR in directly evaluating disability. Our MS population currently includes mainly mild-moderate MS cases, with a small number of patients with severe MS; this is due to the introduction of new therapies and disease-modifying therapies (DMTs) in MS management, which are changing the narrative of the disease. Of note, EDSS is known to be influenced by spinal lesion load [30,31]. Future research should explore more specific cognitive impairment tests, such as Symbol Digit Modalities Test (SDMT), or also digit span or trail-making tests, despite their infrequent use in routine clinical practice.

Furthermore, DIR could have even more relevance in MS follow-up. In fact, routine use of GBCAs is not currently recommended in the follow-up of the disease. This is motivated, in part, by the increased risk of gadolinium retention in patients with MS, who are typically young and will undergo numerous follow-up MRI examinations during their lifetime, and by the fact that the onset of new lesions usually leads to therapeutic switch, regardless of their activity [23]. Use of GBCA in the follow-up may be considered in the setting of suspected clinical relapse or complication of disease-modifying therapy, such as progressive multifocal leukoencephalopathy, or to detect leptomeningeal enhancement. Thus, in this scenario, the use of 3D-DIR sequences, given their ability to highlight cortical lesions related to disability, may lead to a more complete evaluation of disease burden and its prognostic significance even without the need for contrast administration, except when leptomeningeal enhancement is suspected.

**Figure 7:**
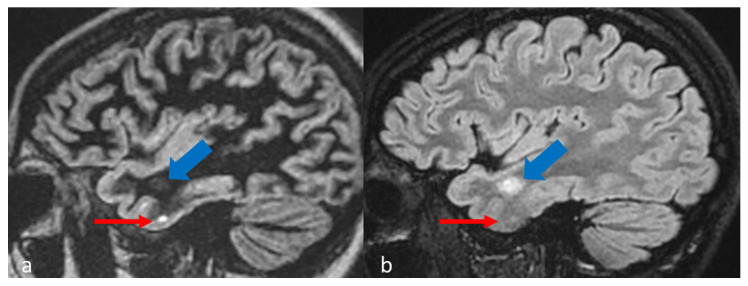
(**a**) The temporal cortical lesion (red arrow) is well detected in the post-contrast sagittal 3D DIR image; (**b**) the same lesion is hardly visualized in the post-contrast sagittal 3D-FLAIR. However, the active subcortical adjacent lesion (blue arrow) resulted suppressed in the post-contrast 3D-DIR.

Another issue to point out is the timing of the DIR sequence within the MRI protocol in MS patients. In our study protocol, 3D-DIR was performed before contrast administration. If these sequences are performed after contrast injection, subcortical active lesions may result suppressed and difficult to detect on DIR images compared to FLAIR or conventional T1-weighted imaging (Figure 7). In fact, although it has been reported that post-contrast DIR is able to detect contrast-enhancing brain lesions in MS, Eichinger et colleagues did not examine native post-contrast DIR, but subtraction images [32]. Thus, pre- and post-contrast DIR should be acquired to obtain a good detection of active lesions in DIR in comparison to classical contrast enhanced T1-weighted imaging, thus resulting in longer duration of the exam without significant clinical benefit for the patient. This highlights the need to follow a rigorous MR protocol in diagnosis and follow-up of MS patients.
**Practical Recommendation**In MS protocol, attention has to be paid to perform DIR before contrast-administration, as post-contrast DIR may suppress active subcortical lesions.

Many previous papers involving DIR analyzed 2D axial DIR and FLAIR sequences with slice thickness of 3–5 mm [7,11,13,19,20,21,22]. Nelson et al. [20] assessed that DIR is prone to artefacts, which may affect the visualization of cortical lesions. However, they used 2D-DIR sequence with section thickness of 3 mm, resulting in partial-volume artefacts, which may mask the presence of small cortical lesions. Instead, we applied 3D sequences, as warmly supported by the current MS guidelines [23]; they may allow a better detection of MS plaques due to their thin slice thickness (<2 mm) and to the chance of obtaining multiplanar reconstructions. Three-dimensional sequences are traditionally considered to require an increased scan time, which has led to consensus recommendations to make them optional for MS [23]. Currently, the accurate choice of acquisition parameters and parallel imaging, such as GeneRalized Autocalibrating Partial Parallel Acquisition (GRAPPA) and Autocalibrating Reconstruction for Cartesian Imaging (ARC) in our study, facilitated their shortening. Multiplanar reformat also waives the need to perform many sequences in different plans. Lespagnol et al. [33] evaluated a Controlled Aliasing In Parallel Imaging Results In Higher Acceleration (CAIPIRINHA)-accelerated DIR sequence, with a scan time of 2 min 23 s, demonstrating that this Fast-DIR improved the detection of juxtacortical MS plaques. Compressed sensing can also be used to reduce scan time of 3D-DIR sequences; moreover, the accelerated DIR proved to be significantly less prone to imaging artifacts [34]. Finally, AI techniques were recently applied to shorten the acquisition time of advanced MRI sequences, allowing the application of advanced techniques, such as 3D-DIR imaging. Currently, Deep Learning can produce images with good quality from under-sampled data, and AI was successfully applied to generate synthetic sequences from already acquired images [35,36,37]. Of particular interest for cortical lesions, Finck et al. used Generative Adversarial Networks (GANs) to generate a DIR sequence from a FLAIR, a T2-weighted and a T1-weighted sequence [37]. Bouman et al. [38] reported that AI-generated DIR images (with U-Net-like convolutional networks) exhibited sensitivity and specificity comparable to conventionally acquired MRI images.

Furthermore, 3D MRI images allow very accurate lesion volume measurements. However, it has traditionally required expert manual reading, which is very time-consuming and prone to intra- and inter-observer variability [39]. Automatic lesion identification and segmentation in 3D sequences with AI and Deep Learning approaches offers the possibility to obtain lesion volume in a fast and reproducible way [36,39,40,41]. In our data set, we did not experiment with automated count, as currently MS lesion segmentation remains an open problem due to the variability of MS lesion appearance and differences in image acquisition [39]. In summary, AI approaches are currently applied for MRI protocol improvement, lesion segmentation, and atrophy assessment, and they will both improve MS diagnosis and will shorten MRI protocols for MS, allowing a more extensive application of advanced techniques such as 3D-DIR in the near future.

Despite the large sample size, our study has some limitations, including the lack of clinical classification of MS subtypes; future investigations should assess the diagnostic value of DIR for confirming dissemination in space in patients with clinically isolated syndrome. Additionally, our analysis was limited to EDSS as the sole clinical score. Further studies should use other clinical scores to overcome the limitations of the EDSS, such as the Multiple Sclerosis Functional Composite (MSFC), and a measure of cognitive function, in order to assess a direct correlation between 3D-DIR lesion count and clinical disability. Another limitation is the lack of inter-reader agreement measurements. Different MR scanners were present in the three institutions: although we carefully standardized our protocols, 3D-DIR and 3D-FLAIR sequences inevitably have some different parameters between the two scanners. In particular, we used 1.5T scanners, although the majority of centers currently have 3T systems; however, the majority of previous papers on this topic used 1.5T scanners, too [7,11,13,19,22,28]. Further research is needed to evaluate the impact of different parameters and image quality in 3D-DIR sequences across various MRI machines.

## 5. Conclusions

The results of this study support the use of 3D-DIR in MS patients, as this sequence demonstrated superior detection of cortical lesions in comparison to 3D-FLAIR. Thus, the direct clinical application of our study comprehends an earlier MS diagnosis using 3D-DIR, as cortical lesions are more easily discernible with this sequence. 3D-DIR, or AI-generated DIR images, should be implemented with little extra time added to the MR routine protocol for the assessment of the cortical lesions, which are correlated to clinical disability, thus fulfilling the requirements of the latest revision of the MS diagnostic criteria.

## Figures and Tables

**Table 1:** MRI sequences parameters.

Repetition Time (ms)	7500	6800	10,000	6000
Echo Time (ms)	310	112	372	105
Inversion Time 1/2 (ms)	3000/450	2650/445	2500	1908
Flip Angle	Variable	Variable	Variable	Variable
Acquisition Matrix	192 × 192	256 × 256	179 × 256	256 × 256
Reconstruction Matrix	192 × 192	256 × 256	256 × 256	ZIP 512
FOV (mm)	280	225	256	256
Slice Number	128	96	144	96
Acquisition Slice Thickness (mm)	1.5	1.8	1	1.8
Reconstruction Slice Thickness (mm)	1	1	1	1
Gap	0	0	0	0
Number of Signals Averaged	1	1	1	1
Parallel Imaging	GRAPPA (iPAT: 2)	ARC	GRAPPA (iPAT: 4)	ARC
Acquisition Time	5 min 39 s	6 min 11 s	6 min 10 s	7 min 9 s
Parameters	3D DIR Siemens	3D DIR GE	3D FLAIR Siemens	3D FLAIR GE

Note: ARC, Autocalibrating Reconstruction for Cartesian Imaging; DIR, Double Inversion Recovery; GRAPPA: GeneRalized Autocalibrating Partial Parallel Acquisition; iPAT: integrated Parallel Acquisition Techniques; min: minutes; ms: milliseconds; s: seconds.

**Table 2:** The demographic and clinical data of final cohort of MS patients.

Number of Patients	278
Age (years) ^1^	47.01 ± 12.668 (18–75)
Gender ^2^	
Female	201 (72.3%)
Male	77 (27.7%)
EDSS ^1^	1.18 ± 1.687 (0–8)

Note: EDSS, expanded disability status scale; ^1^ mean ± SD (range); ^2^ number (%).

**Table 3:** Comparison between 3D-DIR and 3D-FLAIR regarding the number of lesions, mean, and SD in the cohort.

Region	FLAIR			DIR			Z	*p* Value
	No.	Mean	SD	No.	Mean	SD		
Overall burden	6484	23.32	15.200	6601	23.74	16.503	−4089 ^b^	<0.001 *
Infratentorial	543	1.95	2.192	546	1.96	2.198	−0.056 ^c^	0.955
Periventricular WM	2600	9.35	6.196	2610	9.39	6.329	−1.772 ^c^	0.076
Juxtacortical	586	2.11	2.472	613	2.21	2.635	−1.599 ^b^	0.110
Subcortical WM	2596	9.34	8.633	2485	8.94	8.415	–5.814 ^c^	<0.001 *
Cortical	144	0.52	1.029	435	1.56	2.767	–9.502 ^b^	<0.001 *

* Note: ^b^: based on negative ranks; ^c^: based on positive ranks.

**Table 4:** Relationship between cortical affection detected with DIR and FLAIR, and EDSS.

EDSS	Cortical Lesion *n* (%)	No Cortical Lesion *n* (%)	χ^2^	*p* Value
	DIR			
Mild	90 (59.60%)	123 (96.85%)	41.615	<0.001
Moderate	50 (33.10%)	4 (3.15%)		
Severe	11 (7.30%)	0 (0.0%)		
	FLAIR			
Mild	33 (41.25%)	179 (90.40%)	61.006	<0.001
Moderate	37 (46.25%)	18 (9.10%)		
Severe	10 (12.50%)	1 (0.50%)		

**Table 5:** Correlation between EDSS score and the number of MS cortical plaques detected in 3D-DIR and 3D-FLAIR sequences.

MS Plaques	EDSS Score	
	Correlation coefficient ^1^	*p* value (two-tailed)
Cortical lesions in 3D-FLAIR	0.662 **	0.001
Cortical lesions in 3D-DIR	0.874 **	0.000

^1^ Spearman correlation. ** Significant.

## Data Availability

The raw data supporting the conclusions of this article will be made available by the authors on request.
